# Adult Congenital Pulmonary Airway Malformation Presenting With a Large Cystic Lung Lesion: A Case Report

**DOI:** 10.7759/cureus.113094

**Published:** 2026-07-21

**Authors:** Julyan Al-Fori, Dinakar Unnithan, Moyna Dwyer, Amer Saleem

**Affiliations:** 1 Respiratory Medicine, Milton Keynes University Hospital NHS Foundation Trust, Milton Keynes, GBR; 2 Radiology, Milton Keynes University Hospital NHS Foundation Trust, Milton Keynes, GBR; 3 Pathology, Milton Keynes University Hospital NHS Foundation Trust, Milton Keynes, GBR

**Keywords:** adult congenital lung disease, ccam, congenital cystic adenomatoid malformation, congenital pulmonary airway malformation, cpam, cystic lung disease, pet/ct scan, pulmonary hamartoma, thoracic surgery, vats

## Abstract

Congenital pulmonary airway malformation (CPAM) is a rare developmental lung malformation that is usually diagnosed antenatally or during early childhood. Adult presentation is uncommon and may present a significant diagnostic challenge because of overlapping radiological features with other cystic lung diseases and malignancy. We report the case of a 49-year-old man with an incidentally detected large multiseptated cystic lesion occupying almost the entire right upper lobe. CT and PET-CT suggested a congenital cystic lung lesion without evidence of metabolically active malignancy. Following multidisciplinary discussion, the patient underwent video-assisted thoracoscopic wedge resection. Histopathological examination demonstrated benign cystic pulmonary changes without evidence of malignancy. The patient made an excellent postoperative recovery, with complete radiological resolution and objective improvement in pulmonary function. This case highlights the importance of considering CPAM in the differential diagnosis of large cystic pulmonary lesions in adults. Multidisciplinary assessment and surgical resection remain essential for establishing a definitive diagnosis and achieving favorable clinical outcomes.

## Introduction

Congenital pulmonary airway malformation (CPAM) is a rare congenital dysplastic lung malformation characterized by abnormal bronchial development and localized glandular overgrowth [1,2]. It accounts for approximately 25% of all congenital lung malformations and up to 95% of congenital cystic lung lesions [1,2]. Diagnosis can be established antenatally using prenatal ultrasonography and confirmed postnatally with imaging modalities such as chest radiography and MRI. While most cases are diagnosed during the prenatal or neonatal period, a small number present in childhood, with even fewer diagnosed in adulthood. In older children and adults, CPAM is often identified incidentally or following recurrent respiratory infections. Factors associated with poorer outcomes include hydrops fetalis, microcystic CPAM, and larger lesion size [3,4]. CPAM is categorized into five types, ranging from type 0 to type IV, based on the size, appearance, and origin of the affected pulmonary regions. CPAM is known to have the potential for malignant transformation [3,4].

Given the risks of recurrent infection, progressive lung damage, and malignant transformation, surgical resection remains the treatment of choice for adult CPAM, even in asymptomatic patients. The extent of resection, whether segmentectomy or lobectomy, is determined by the size and anatomical location of the lesion. However, despite an increasing number of case reports and small case series, large cohort studies systematically evaluating the clinical presentation, radiological features, pathological subtypes, and surgical outcomes of adult CPAM remain limited [5].

## Case presentation

**Figure 1:**
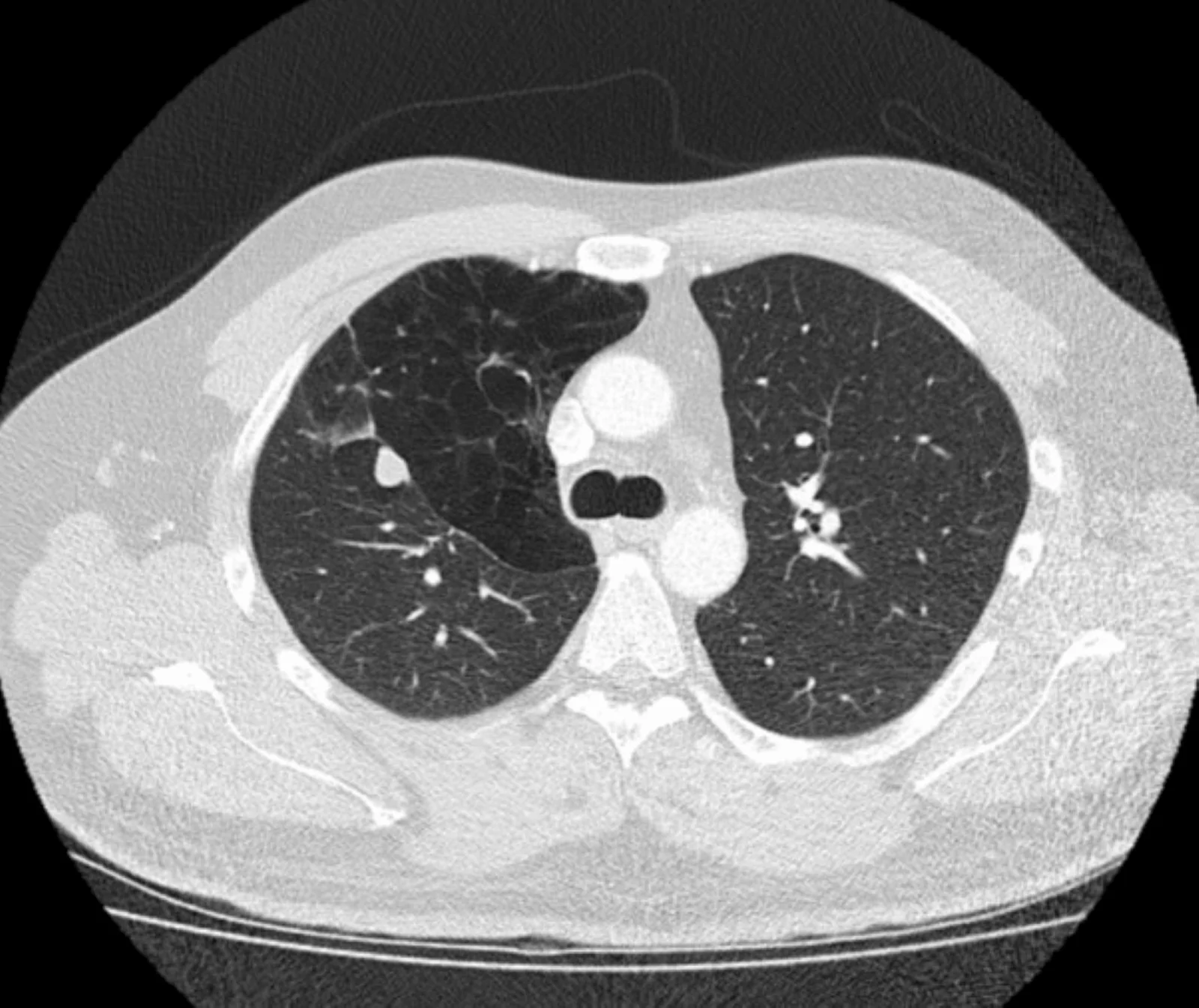
Axial contrast-enhanced CT scan demonstrating a large multicystic lesion of the right upper lobe.

A 49-year-old man was referred to the respiratory service following the incidental identification of extensive cystic abnormalities involving the right upper lobe on CT imaging performed in June 2019. His past medical history included renal stone disease in 2015, benign prostatic hyperplasia, and fatty liver disease. He was an ex-smoker. Contrast-enhanced CT of the thorax demonstrated a large multiseptated cystic lesion occupying almost the entirety of the right upper lobe, measuring approximately 10 × 9 × 15 cm (Figure 1).

**Figure 2:**
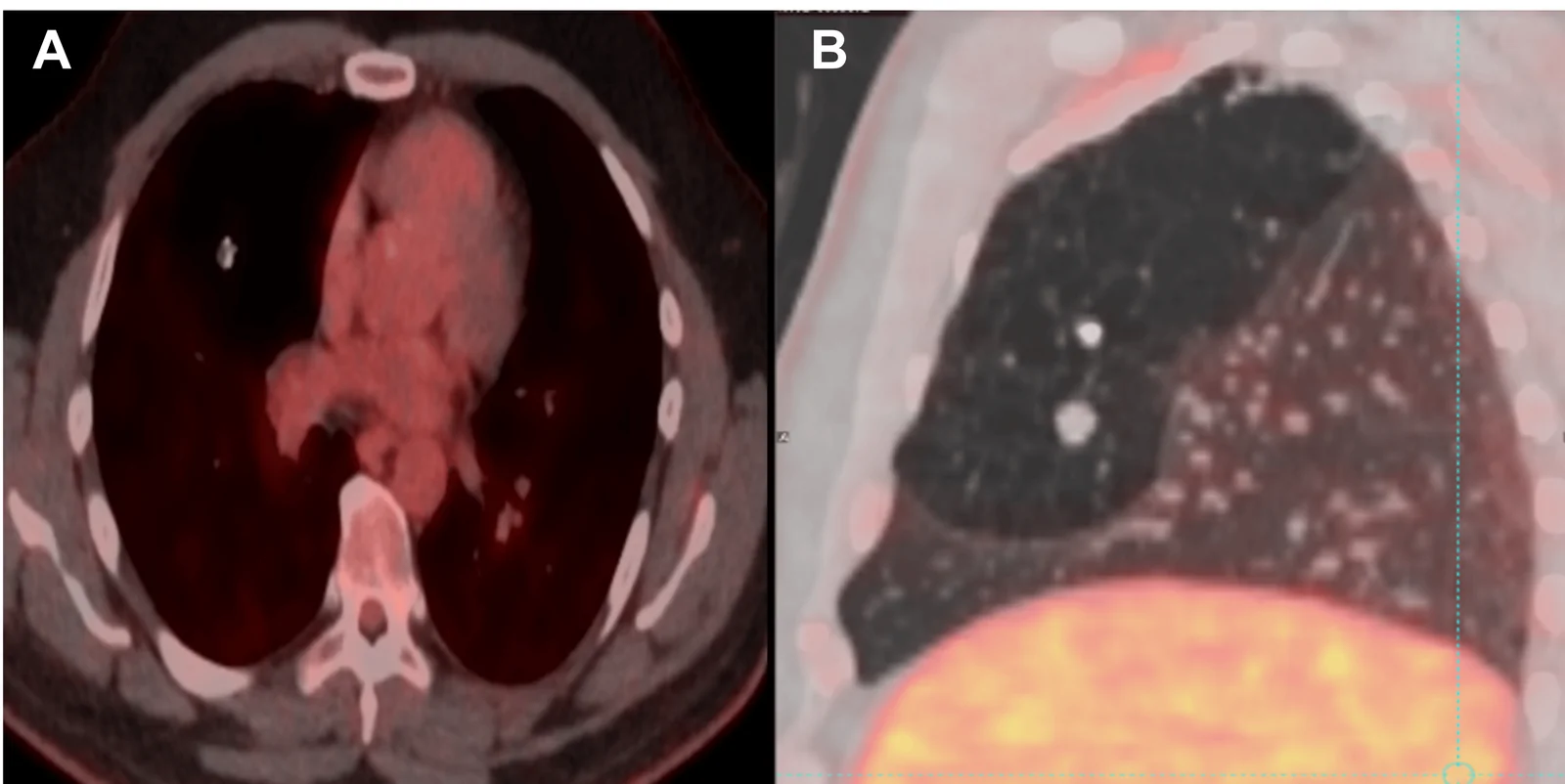
(A) Axial fused PET-CT demonstrating minimal FDG uptake within the right upper lobe cystic lesion. (B) PET-CT showing the cystic lesion with calcified nodules.

No mediastinal, hilar, or subcarinal lymphadenopathy was identified. Importantly, there were no solid soft tissue nodules or fluid levels within the cystic spaces. The radiological appearances were considered strongly suggestive of CPAM presenting in adulthood. The case was subsequently reviewed at the lung multidisciplinary team (MDT) meeting following PET-CT evaluation. PET-CT showed an overinflated right upper lobe containing multiple thin-walled cysts and partially calcified nodules with minimal FDG avidity (maximum standardized uptake value (SUVmax) 0.3) and no evidence of metabolically active disease elsewhere (Figure 2A, 2B). Given the lesion size, diagnostic uncertainty, and recognized malignant potential associated with congenital cystic pulmonary lesions, surgical management was recommended [6-8]. The patient underwent right-sided video-assisted thoracoscopic surgical wedge resection in January 2020. Given the localized nature of the lesion and the absence of suspicious invasive malignant features on imaging, a lung-preserving minimally invasive surgical approach was considered appropriate.

**Figure 3:**
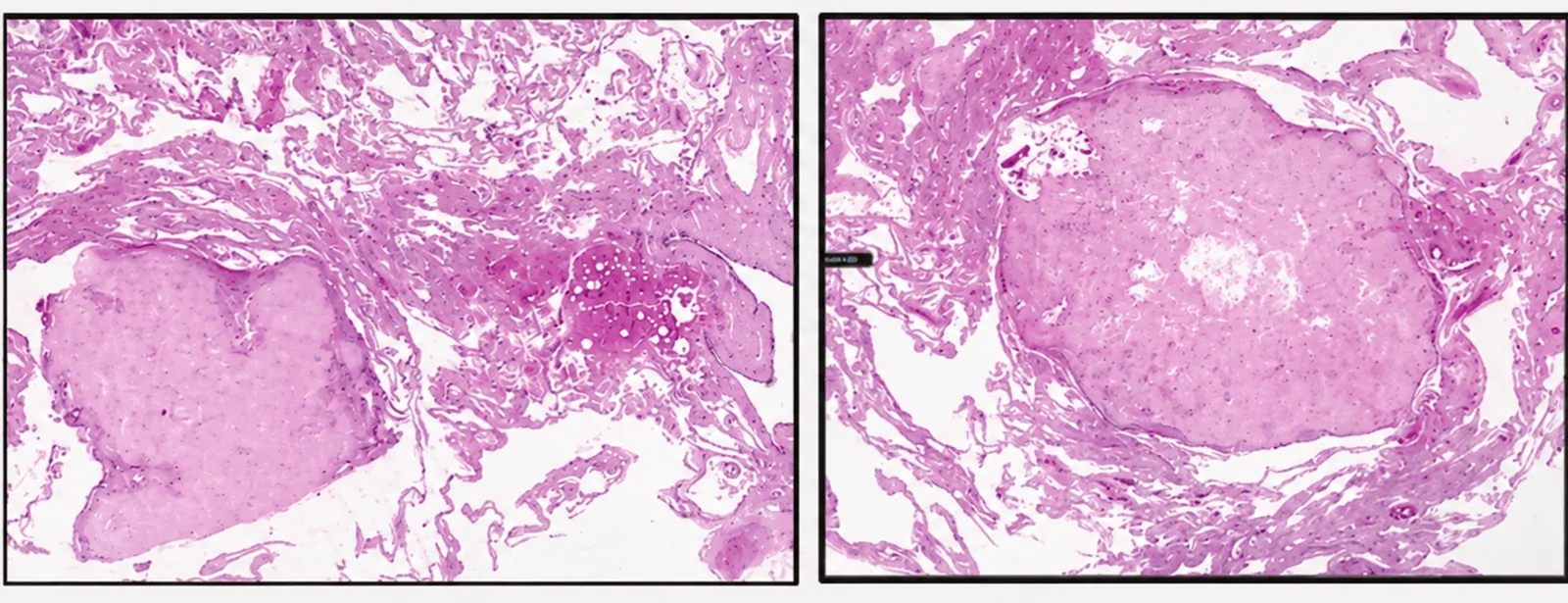
Histopathological examination demonstrating pulmonary hamartomatous features with mature chondroid tissue. Hematoxylin and eosin-stained sections demonstrating mature chondroid tissue within the cyst wall with focal calcification and ossification. No granulomatous inflammation, immature mesenchymal proliferation, or malignant transformation is identified.

Histopathological examination demonstrated a large cystic lesion associated with three well-circumscribed nodules composed predominantly of mature chondroid tissue with admixed loose mesenchymal stroma and adipose tissue. The cyst wall showed fibroelastic tissue with focal calcification and ossification. No granulomatous inflammation, immature mesenchymal proliferation, or malignant transformation was identified. The overall appearances were reported as multiple pulmonary hamartomas with secondary cystic change, and complete excision was achieved (Figure 3).

**Figure 4:**
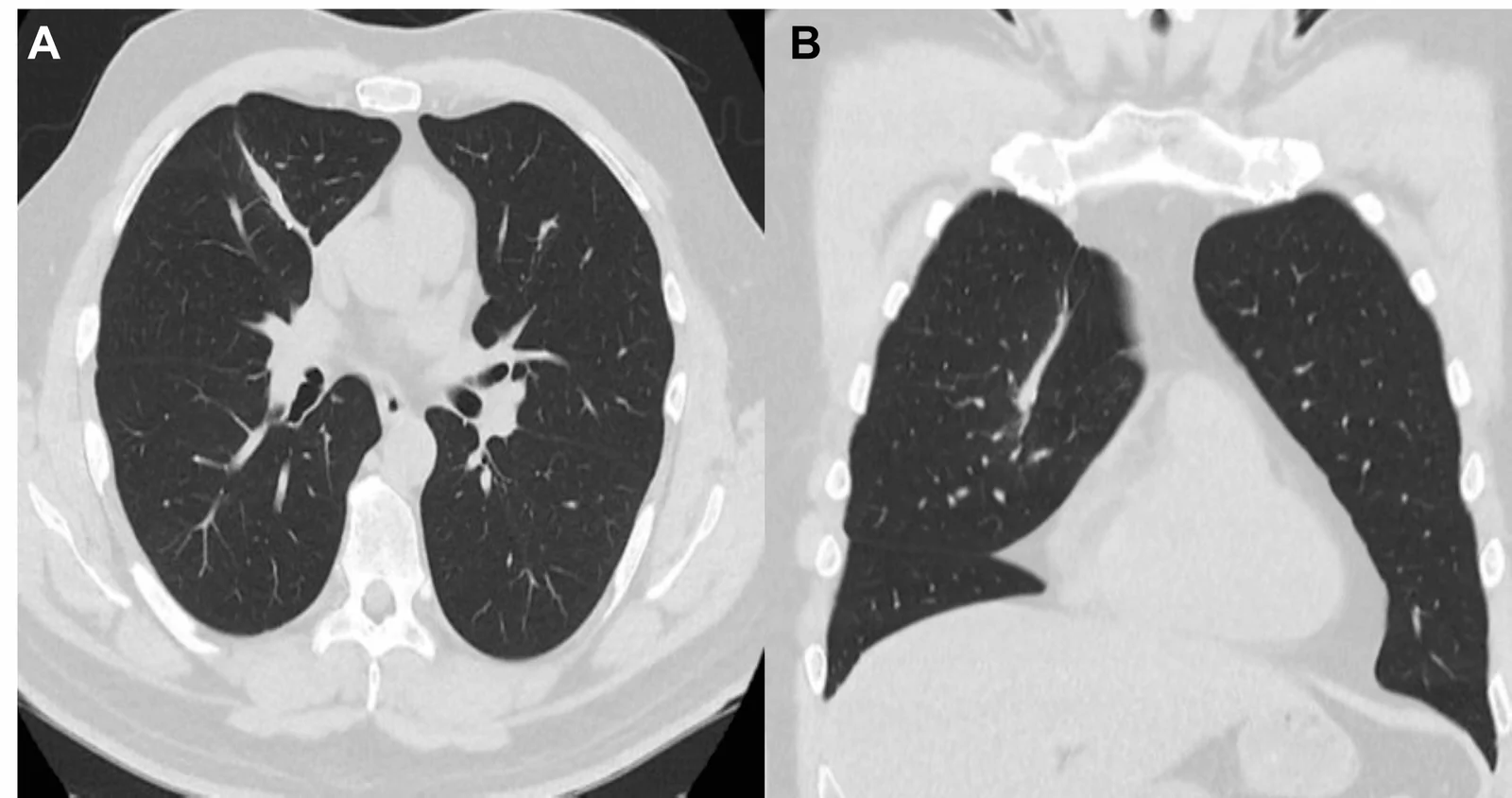
Postoperative CT of the chest following wedge resection. Axial (A) and coronal (B) high-resolution CT images demonstrating postoperative changes in the right upper lobe with linear scarring and no residual cystic lesion or suspicious pulmonary nodule.

Postoperatively, the patient made an excellent respiratory recovery and remained clinically stable. At outpatient follow-up, he denied cough, sputum production, hemoptysis, chest pain, constitutional symptoms, or recurrent respiratory tract infections. Oxygen saturation was 100% on room air. Surveillance high-resolution CT imaging was performed, which demonstrated expected postoperative changes within the right upper lobe with linear scarring. No residual cystic pulmonary abnormality or suspicious nodular lesion was identified (Figure 4).

Pulmonary function testing demonstrated objective postoperative physiological improvement. Forced expiratory volume in one second (FEV1) was 3.79 L (89% predicted), forced vital capacity (FVC) was 5.04 L (94.5% predicted), and the FEV1/FVC ratio was 75%. Residual volume improved from 131% predicted preoperatively to 75% predicted postoperatively, suggesting improved pulmonary function and reduced regional hyperinflation following resection of the large cystic lesion. Given the favorable postoperative clinical, radiological, and physiological outcomes, the patient was discharged from respiratory follow-up.

## Discussion

CPAM is a rare congenital developmental anomaly of the lung resulting from abnormal branching morphogenesis of the terminal bronchioles during fetal development. This malformation leads to the formation of cystic, nonfunctioning lung tissue that does not participate in gas exchange [9,10]. Although CPAM is uncommon, it accounts for approximately 25% of all congenital lung malformations. Clinical manifestations include recurrent respiratory tract infections, pneumothorax, hemoptysis, and dyspnea. Importantly, CPAM also carries a risk of malignant transformation [7,11].

Differentiating CPAM from other cystic pulmonary diseases on imaging remains challenging because of considerable overlap in radiological features. CPAM may present radiologically as unilocular or multilocular cysts, cavitary lesions, or mixed cystic-solid masses and can occasionally mimic bronchiectasis, lung abscesses, or even malignancy. Recent studies have also highlighted that adult CPAM may coexist with other pulmonary abnormalities, including pulmonary sequestration, chronic infection, and malignant tumors such as adenocarcinoma in situ, mucinous adenocarcinoma, and carcinoid tumors [4,6,8]. These findings further emphasize the importance of thorough histopathological evaluation following surgical resection. Given the risks of recurrent infection, progressive lung damage, and malignant transformation, surgical resection remains the treatment of choice for adult CPAM, even in asymptomatic patients. The extent of resection, whether by segmentectomy or lobectomy, is determined by the size and location of the lesion [4]. In our case, the presence of extensive multiseptated cystic architecture, together with the absence of significant soft tissue nodularity, strongly favored a congenital cystic lesion rather than a cavitating malignancy.

CPAM is classified into five subtypes (Types 0-4) according to the widely accepted Stocker classification, based on histological and pathological characteristics. This classification provides important insight into the embryological origin, clinical presentation, and prognosis of each subtype [6,9,10]. Type 0 is the rarest form of CPAM and originates from the tracheobronchial tree. It is characterized by small, firm lungs with a solid appearance and no cyst formation. Histologically, Type 0 lesions are lined by ciliated pseudostratified columnar epithelium and contain cartilage and submucosal glands, resembling normal bronchial tissue. Owing to severe pulmonary hypoplasia and diffuse lung involvement, this subtype is generally incompatible with life [6]. Type 1 CPAM is the most common subtype, accounting for approximately 60-70% of cases. It typically consists of one or more large cysts measuring >2 cm in diameter, which are often multilocular. Histologically, the cysts are lined by ciliated pseudostratified columnar epithelium and may contain mucus-producing goblet cells. The cyst walls contain smooth muscle but usually lack cartilage. Type 1 lesions are associated with an excellent prognosis, and surgical resection is generally curative. Rare cases of malignant transformation, particularly to mucinous bronchioloalveolar carcinoma, have nevertheless been reported [6].

Type 2 CPAM is characterized by multiple small cysts measuring <2 cm, producing a spongy appearance. Microscopically, the cysts resemble bronchioles and are lined by cuboidal to columnar epithelium, with smooth muscle present within the cyst walls. This subtype is frequently associated with other congenital anomalies, including renal and cardiac defects, and its prognosis largely depends on these associated abnormalities [6]. Type 3 CPAM usually presents as a bulky solid mass with minimal cystic change and commonly involves an entire pulmonary lobe. Histologically, it consists of small bronchiole-like structures and alveolar ducts lined by cuboidal epithelium. The associated mass effect and limited cyst formation often result in severe respiratory distress during the neonatal period, contributing to a poorer prognosis [6]. Type 4 CPAM is characterized by large, thin-walled peripheral cysts, often exceeding 10 cm in diameter, which are lined by flattened alveolar-type epithelium resembling distal acinar structures. This subtype may present later in childhood or adulthood and carries a higher risk of malignant transformation, particularly to pleuropulmonary blastoma. Consequently, surgical resection is generally recommended even in asymptomatic patients [6,8].

Pathologically, CPAMs are hamartomatous lesions composed of cystic and adenomatous elements arising from tracheal, bronchial, bronchiolar, or alveolar tissue. Large lesions may compromise alveolar growth and development through compression of the surrounding normal lung parenchyma. Histologically, the cysts are lined by epithelial cells corresponding to their site of origin, ranging from pseudostratified ciliated columnar epithelium in Type 1, to cuboidal epithelium in Types 2 and 3, and squamous epithelium in Type 4 [9].

A multidisciplinary approach involving respiratory physicians, thoracic surgeons, radiologists, and pathologists is essential for guiding the diagnosis and management of these complex cases. In our patient, MDT discussion was instrumental in balancing diagnostic uncertainty against the potential risk of malignancy associated with congenital cystic pulmonary lesions. Surgical resection provides both definitive diagnosis and therapeutic benefit [9,11]. Although lobectomy is commonly advocated for extensive disease, limited resection may be appropriate in selected patients with localized lesions and favorable anatomical characteristics. Video-assisted thoracoscopic surgery offers several advantages over open thoracotomy, including reduced postoperative pain, shorter hospital stay, lower postoperative morbidity, and faster recovery [8,11].

Interestingly, our patient demonstrated objective physiological improvement following surgery, particularly a marked reduction in residual volume. This most likely reflects improved pulmonary mechanics after removal of the large cystic lesion and resolution of regional hyperinflation. Similar improvements in postoperative lung function have been reported in selected surgical series of patients with congenital cystic pulmonary lesions [11].

This case highlights several important clinical learning points. First, CPAM should remain an important differential diagnosis when evaluating large cystic pulmonary lesions in adults. Second, congenital pulmonary abnormalities may remain clinically silent for decades before being detected incidentally on imaging. Third, multidisciplinary evaluation is essential when radiological appearances overlap with malignant pathology. Finally, surgical management can provide excellent long-term clinical, radiological, and physiological outcomes.

## Conclusions

Adult presentation of CPAM is rare and can pose significant diagnostic challenges due to its overlap with other cystic pulmonary diseases and malignant conditions. This case highlights the unusual coexistence of CPAM with a pulmonary hamartoma, an association that, to our knowledge, has not previously been reported. Recognition of this entity, along with appropriate multidisciplinary evaluation, is essential for guiding optimal management.
